# Serum Uric Acid Levels as a Risk Stratification Tool in Hypertensive Disorders of Pregnancy: A Retrospective Study

**DOI:** 10.7759/cureus.65395

**Published:** 2024-07-25

**Authors:** Ritu Singh, Mukta Agarwal, Avinash K Singh, Sudwita Sinha, Hemali H Sinha, Monika Anant

**Affiliations:** 1 Obstetrics and Gynaecology, All India Institute of Medical Sciences Patna, Patna, IND; 2 Radiodiagnosis, All India Institute of Medical Sciences Patna, Patna, IND

**Keywords:** neonatal outcome, maternal outcome, risk stratification, eclampsia, preeclampsia, gestational hypertension, uric acid

## Abstract

Background: Worldwide, hypertensive disorders of pregnancy (HDP) are among the leading causes of maternal and fetal morbidity and mortality. Serum uric acid is a test that can evaluate the severity of HDP and the associated maternal and fetal morbidity and mortality.

Aim: To examine the relationship between maternal serum uric acid levels and the severity of HDP and overall pregnancy outcomes.

Material and methods: A retrospective study was conducted on women with a gestational age > 20 weeks and BP >140/90 mmHg over three years. A total of 134 patients were included in the study. Patients with chronic hypertension, hyperuricemia without hypertension, and other major illnesses were excluded. Data were collected from medical records, including age, gravida, parity, weight, height, gestational age, blood pressure at admission, urine albumin, and serum uric acid levels.

Results: Of the 134 enrolled women with HDP, 76 had gestational hypertension, 41 had preeclampsia, and 17 had eclampsia. Mean uric acid levels in mg/dL were 6.06±1.651, 6.20±0.824, and 7.38±1.26 in gestational hypertension, preeclampsia, and eclampsia, respectively, which was a significant association (p=0.002). Mean uric acid in mg/dL was 5.86±1.27 in intensive care unit (ICU) patients compared to 6.45±1.39 in ward patients (p=0.015). There was a significantly increased risk of ICU admission and preterm delivery (r=-0.401, p<0.001) in patients with elevated uric acid levels. There was a significantly increased risk of low-birth-weight babies with elevated uric acid levels (r=-0.278, p=0.001). However, there was no statistically significant increased risk of newborn intensive care unit admissions (p=0.264) with elevated uric acid levels.

Conclusion: Serum uric acid levels vary significantly in HDP and were found to be elevated in severe preeclampsia and eclampsia. It can be considered for risk stratification in HDP based on disease severity; however, its role in determining outcomes is debatable. Using serum uric acid levels in predictive models along with known biomarkers may determine its possible additional value in disease prediction and severity.

## Introduction

Hypertensive disorders of pregnancy (HDP) are among the leading causes of maternal and fetal morbidity and mortality worldwide [1]. Globally, there was a 10.92% (16.30-18.08 million) increase in the incidence of HDP from 1990 to 2019, with the highest regional incidence of 3.84 million in South Asia. The number of deaths due to HDP also increased by 30.05% from 1990, reaching approximately 27.83 thousand in 2019 [2].

The poor maternal and fetal outcomes in HDP are due to the lack of a single specific test that can identify pregnant women at risk for HDP [3]. One such test is serum uric acid (SUA), which can evaluate the severity of HDP and the associated maternal and fetal morbidity and mortality.

SUA levels increase in HDP due to tissue breakdown, acidosis, decreased renal clearance, and increased activity of the xanthine oxidase/dehydrogenase enzyme [4,5]. In the first trimester, uric acid levels are <3 mg/dL due to uricosuria and increased renal blood flow (estrogen effect). In the third trimester, it reaches 4-5 mg/dL at term [6].

The use of SUA in HDP was first described in 1917 [7], and it is now also considered for HDP severity [6-9]. Further, it was reported that, in women with HDP, the increase in uric acid occurs well before hypertension and proteinuria develop [4,10], and at-risk HDP women have mildly elevated uric acid levels compared to those in normal pregnancies in the first trimester [6,11].

Despite these findings, recent meta-analyses do not recommend using uric acid to predict adverse maternal and fetal outcomes [12]. Hence, this study evaluated the use of SUA as a risk stratification tool in HDP. The aim of this study was to examine the relationship between maternal SUA levels and the severity of HDP and overall pregnancy outcomes.

## Materials and methods

Study design

This is a retrospective study from a retrospective chart review.

Study setting

The study was conducted in the Department of Obstetrics and Gynaecology, All India Institute of Medical Sciences (AIIMS), Patna, Bihar, from September 2016 to August 2019, using medical record papers, after approval from the Institutional Ethics Committee (Letter no.: AIIMS/Pat/IEC/2021/787).

Study participants

Pregnant women admitted with hypertensive disorders of pregnancy with a gestational age ≥20 weeks and blood pressure ≥140/90 mmHg were included in the study. Exclusion criteria included pregnant women with chronic hypertension, hyperuricemia without hypertension, gout, diabetes mellitus, a history of renal, liver, or cardiovascular illness, epilepsy, a urinary tract infection, and a history of smoking, alcohol, or substance abuse.

Variables 

The data were collected from patient files retrieved from the Medical Records Department using pre-structured proformas, including parameters such as age, gravida, parity, weight, height, gestational age, blood pressure at admission, urine albumin, SUA levels, and neonatal weight.

The quantitative variables are as follows. From the height and weight of the participants, body mass index (BMI) was calculated as weight in kg/height in meter x height in meter (kg/m^2^). It was then tabulated as normal (18.5-24.9 kg/m^2^), overweight (25-29.9 kg/m^2^), and obese (>30 kg/m^2^). Gestational age at delivery was classified into three groups - early preterm (28-34 weeks), late preterm (34-37 weeks), and term (>37 weeks).

Outcome measures

The primary outcome was the correlation between maternal uric acid levels and hypertensive disorders of pregnancy. The secondary outcomes are as follows: (1) Association of SUA levels with gestational age at delivery. (2) Association of SUA levels with age, parity, BMI, and mode of delivery. (3) Correlation between uric acid and maternal outcome in terms of ICU admission and fetal outcome in terms of NICU admission and birth weight of neonates.

Hypertensive disorders of pregnancy included gestational hypertension, preeclampsia, and eclampsia, which were defined as follows.

Definitions

Gestational hypertension: A clinical diagnosis defined by the new onset of hypertension (systolic blood pressure ≥140 mmHg and/or diastolic blood pressure ≥90 mmHg) on two occasions at least four hours apart after 20 weeks of gestation in the absence of proteinuria or new signs of end-organ dysfunction [13].

Preeclampsia: Systolic blood pressure of 140 mmHg or more or diastolic blood pressure of 90 mmHg or more on two occasions at least four hours apart after 20 weeks of gestation in a woman with previously normal blood pressure and proteinuria of 300 mg or more per 24-hour urine collection or protein/creatinine ratio of 0.3 mg/dL or more or dipstick reading of 2+, or, in the absence of proteinuria, new-onset hypertension with the new onset of any of the following: thrombocytopenia (platelet count less than 100,000 x 10^9^/L), renal insufficiency: serum creatinine concentrations greater than 1.1 mg/dL or a doubling of the serum creatinine concentration in the absence of other renal disease, impaired liver function: elevated blood concentrations of liver transaminases to twice normal concentration, pulmonary edema, new-onset headache unresponsive to medication and not accounted for by alternative diagnoses, or visual symptoms [13,14].

Severe preeclampsia: Systolic blood pressure of 160 mmHg or more or diastolic blood pressure of 110 mm Hg or more on two occasions at least four hours apart, thrombocytopenia, and impaired liver function that is not accounted for by alternative diagnoses and as indicated by abnormally elevated blood concentrations of liver enzymes (to more than twice the upper limit normal concentrations), or by severe persistent right upper quadrant or epigastric pain unresponsive to medications, renal insufficiency (serum creatinine concentration more than 1.1 mg/dL or a doubling of the serum creatinine concentration in the absence of other renal diseases), pulmonary edema, new-onset headache unresponsive to medication and not accounted for by alternative diagnoses, and visual disturbances [13,14].

Eclampsia: A convulsive condition associated with preeclampsia [14]. Eclampsia is defined by new-onset tonic-clonic, focal, or multifocal seizures in the absence of other causative conditions such as epilepsy, cerebral arterial ischemia and infarction, intracranial hemorrhage, or drug use [14].

Data source measurement

The reference levels considered for normal serum uric acid levels were 2.5-5.6 mg/dL in non-pregnant females, 2-4.2 mg/dL in pregnant first-trimester patients, 2.4-4.9 mg/dL in pregnant second-trimester patients, and 3.1-6.3 mg/dL in pregnant third-trimester patients [15]. The completed weeks of gestation were taken as the period of gestation. One and two previous cesarean sections were grouped as elective repeat cesarean sections.

Biases

Sampling bias was not addressed as random sampling was not done and the convenience sampling technique was used. However, reviewer bias was addressed by blinding the data collector to the purpose of the study.

Sample size

A pilot study was conducted, and 30 random medical record sheets of discharged pregnant women were taken out from the Medical Records Department, out of which two patients were found to be hypertensive. Taking a rounded-up percentage of 6 (i.e., 0.06 as proportion, 95% confidence interval, and 0.05 as margin of error), the sample size calculated was 155. There were a total of 1,716 deliveries during the above-mentioned duration of the study period of three years, of which 156 patients had hypertensive disorders of pregnancy. Out of 156 patients, nine had chronic hypertension and were hence excluded from the study. In 19 patients, SUA values were not attached to the file. Efforts were made to retrieve uric acid values from the hospital information system, which is software for keeping medical records, and from the microbiology department. Despite these efforts, the SUA values of only six patients could be retrieved. Thirteen patients whose SUA values could not be retrieved were also excluded from the study. Hence, a total of 134 patients were included in the study.

Statistical analysis

Statistical software Jamovi 2.3.28 solid version (https://www.jamovi.org) was used for data analysis. SUA levels were presented as mean and standard deviation. Socio-demographic features were presented as frequency and percentage. For comparing SUA levels with maternal and fetal outcomes, a one-way ANOVA/independent sample t-test was applied, with a p-value <0.05 considered statistically significant.

Ethical considerations

The study was conducted after approval from the Institutional Ethics Committee of All India Institute of Medical Sciences (Letter no.-AIIMS/Pat/IEC/2021/787).

## Results

Of the 134 patients with HDP included in the study, 76 had gestational hypertension, 41 had preeclampsia, and 17 had eclampsia. Table 1 shows the socio-demographic features. BMI was calculated from pre-delivery weight and height, and there were no underweight patients.

**Table 1:** Socio-demographic features.

Age (in years)	Gestational Hypertension (%), N=76	Preeclampsia (%), N=41	Eclampsia (%), N=17
<20–25	20 (14.9%)	20 (14.9%)	0 (0%)
26–30	31 (23.1%)	5 (3.7%)	11 (8.2%)
31–35	2 0 (14.9%)	16 (11.9%)	6 (4.5%)
36–40	5 (3.7%)	0 (0.0%)	0 (0.0%)
Parity	Gestational Hypertension (%), N=76	Preeclampsia (%), N=41	Eclampsia (%), N=17
Primipara	45 (33.6%)	26 (19.4%)	6 (4.5%)
Multipara	31 (23.1%)	15 (11.2%)	11 (8.2%)
Body Mass Index (BMI)	Gestational Hypertension (%), N=76	Preeclampsia (%), N=41	Eclampsia (%), N=17
Normal (18.5–24.9)	20 (14.9%)	11 (8.2%)	5 (3.7%)
Overweight (25–29.9)	31 (23.1%)	5 (3.7%)	6 (4.5%)
Obese (>30)	25 (18.7%)	25 (18.7%)	6 (4.5%)
Gestational age at admission (weeks)	Gestational Hypertension (%), N=76	Preeclampsia (%), N=41	Eclampsia (%), N=17
≥28 to <34	5 (3.7%)	6 (4.5%)	6 (4.5%)
≥34 to <37	26 (19.4%)	15 (11.2%)	5 (3.7%)
≥37 to <42	45 (33.6%)	20 (14.9%)	6 (4.5%)

The mean age of the participants was 28.3±4.62 years (21-39 years). The mean BMI was 37.4±4.57 kg/m^2^ (30.2-47.7), and the mean gestational age at admission was 36.7±2.29 weeks (30-39).

Vaginal deliveries, primary cesarean sections, and repeat cesarean sections were performed on 20, 72, and 42 patients, respectively. Eighty-six patients received care in a normal ward, whereas 48 required ICU care. Thirty-four newborns needed newborn intensive care unit (NICU) care. The indications for NICU admission of newborns included prematurity (11), respiratory distress syndrome (9), transient tachypnea of newborns (5), hyperbilirubinemia (4), meconium aspiration syndrome (2), hypoglycemia (2), and neonatal sepsis (1). In contrast, 100 babies did not require NICU admission. Table 2 shows the association of uric acid levels with hypertensive disorders, mode of delivery, maternal outcomes, and fetal outcomes.

**Table 2:** Frequency of cases and association of uric acid with hypertensive disorders, mode of delivery, maternal outcomes, and fetal outcome. *One-way ANOVA, **Independent sample T-test

Hypertensive Disorder	Frequency of cases, n (N=134)	Uric Acid mg/dL (mean ± standard deviation)	p-value for uric acid
Gestational Hypertension	76	6.06±1.651	0.002*
Preeclampsia	41	6.20±0.824	
Eclampsia	17	7.38±1.26	
Mode of Delivery	Frequency of cases, n (N=134)	Uric Acid mg/dL (mean ± standard deviation)	p-value for uric acid
Vaginal Delivery	20	6.60±1.41	0.474*
Primary Cesarean Section	72	6.16±1.48	
Elective Repeat Cesarean Section	42	6.30±1.44	
Maternal Outcome	Frequency of cases, n (N=134)	Uric Acid mg/dL (mean ± standard deviation)	p-value for uric acid
Ward Care	86	5.86±1.27	0.015**
ICU (Intensive Care Unit) Care	48	6.45±1.39	
Fetal Outcome	Frequency of cases, n (N=134)	Uric Acid mg/dL (mean ± standard deviation)	p-value for uric acid
Newborn intensive care unit (NICU) admission is not required	100	6.15±1.43	0.264**
NICU Admission Required	34	5.85±1.03	

Table 3 shows the Pearson correlation between SUA and age, BMI, parity, gestational age at delivery, and neonatal birth weight.

**Table 3:** Pearson correlation between serum uric acid and different parameters.

Parameters	Pearson Correlation	P-value
Age	0.039	0.654
BMI	0.183	0.034
Parity	-0.96	0.272
Gestational age at delivery	-0.401	<0.001
Neonate birth weight	-0.278	0.001

## Discussion

The present study observed that maternal uric acid levels profoundly influenced the severity of hypertensive disorders of pregnancy and subsequent maternal outcomes. As the severity of hypertension increased, SUA levels also increased. The mean values of uric acid were 6.06±1.651 mg/dL in gestational hypertension, 6.20±0.824 mg/dL in preeclampsia, and 7.38±1.26 mg/dL in eclampsia. Similar results were shown in a North Indian study conducted by Kumar et al. on women ≥34 weeks of gestation, where uric acid levels in gestational hypertension, preeclampsia, and eclampsia were 5.47±1.93 mg/dL, 6.72±2.15 mg/dL, and 8.71±2.97 mg/dL, respectively [16]. Another study by Yuan et al. evaluated uric acid levels in preeclamptic women and normal controls, finding that uric acid levels were significantly elevated in patients with preeclampsia (3.21±0.72 μmol/L vs. 7.65±0.81 μmol/L) [17]. Additionally, a study by Koley et al. indicated a strong association of SUA levels with the severity of the disease, showing significant elevation of uric acid in severe preeclampsia (7.07±0.22 mg/dL vs. 4.48±0.76 mg/dL, p<0.05) but not in mild preeclampsia (4.48±0.76 mg/dL vs. 5.05±1 mg/dL, p<0.05) [18]. A recent study also reported a strong correlation between increasing maternal SUA and the severity of preeclampsia [19]. Studies have also reported that blood pressure is positively correlated with uric acid levels [20], meaning that, as blood pressure increases, uric acid levels also rise.

In our study, the period of gestation at the diagnosis of preeclampsia and eclampsia was mostly <37 completed weeks, whereas gestational hypertension was diagnosed mostly at or near term. Other studies have also quoted that, in severe HDP, the period of gestation at diagnosis was earlier than in gestational hypertension (p<0.001) [21-23].

Our study did not find any significant association between uric acid levels and the mode of delivery, which is consistent with findings from another study conducted by Le et al. [24]. However, other studies have reported a higher rate of cesarean sections with higher uric acid levels [12].

Uric acid levels were also found to be significantly correlated with adverse maternal outcomes requiring ICU care in our study. Similar results were reported by various other studies, which demonstrated a strong correlation between elevated maternal SUA levels and adverse maternal outcomes [4,6,25]. However, recent studies have shown that, although there are differences in uric acid levels between women receiving ICU care and those receiving ward care with uric acid, they are not significant [26-28].

Although a few previous studies have shown adverse neonatal outcomes in women with high uric acid levels [16,29], we did not find a significant association between SUA levels and NICU admission of neonates.

In our study, there was a statistically significant negative Pearson correlation between uric acid levels and gestational age at delivery (r=-0.401, p<0.001), indicating that, as uric acid levels increase, the gestational age at delivery decreases, leading to preterm delivery. These findings are similar to those of a study conducted by Le et al. [24]. Additionally, in our study, neonatal birth weight was significantly negatively correlated with uric acid levels (r=-0.278, p=0.001), meaning that low-birth-weight babies were delivered to patients with higher SUA levels. Similar results were found in another study on intrauterine growth restriction and uric acid (OR: 7.188, 95% CI: 3.592-14.382) [24].

Limitations

There are certain limitations to our study. First, as the study was conducted in a tertiary care center with mostly complicated referred cases, its prevalence cannot be generalized to the broader population. Secondly, as this was a retrospective study, preconception, and conceptional data could not be collected, which limited the evaluation of maternal risks. Thirdly, only the uric acid value at admission was used; repeated measures of uric acid were not taken. Further research with prospective studies is required to determine causal inference and the cutoff value of uric acid levels that determine severity.

## Conclusions

The present study observed that SUA levels vary significantly in HDP, being higher in severe preeclampsia and eclampsia. Preeclampsia and eclampsia present at earlier weeks of gestation. In contrast, gestational hypertension presents mostly at term. There is an increased risk of ICU admission and preterm delivery in patients with high uric acid levels. There is also an increased risk of delivering low-birth-weight babies with high uric acid levels. However, NICU admissions were not statistically significant in relation to uric acid levels. Therefore, SUA levels can be considered for risk stratification in HDP for disease severity, but their role in determining outcomes is debatable. Using SUA in predictive models, along with known biomarkers, will help determine its possible additional value in disease prediction and severity.
